# Human albumin and 6% hydroxyethyl starches (130/0.4) in cardiac surgery: a meta-analysis revisited

**DOI:** 10.1186/s12893-022-01588-x

**Published:** 2022-04-12

**Authors:** Christian J. Wiedermann

**Affiliations:** Institute of General Practice and Public Health, Claudiana College of Health Professions, Lorenz Böhler Street 13, 39100 Bolzano, Italy

**Keywords:** Adverse effects, Colloids, European Medicines Agency, Plasma substitutes, Renal dysfunction, Surgery

## Abstract

**Background:**

A meta-analysis of randomized controlled trials was recently published in BMC Surgery that compared the use of human albumin with 6% hydroxyethyl starches 130/0.4 for cardiopulmonary bypass prime and perioperative fluid management in pediatric and adult cardiac surgery patients. The two plasma expanding solutions are described as equivalent for efficacy and safety outcomes, and, on that basis, the preferential use of hydroxyethyl starches 130/0.4 was recommended for economic reasons because of the higher unit costs of human albumin solutions.

**Results:**

In addition to the fact that trials were mostly small, single-center studies and the number of total participants was low, making the meta-analysis underpowered for several outcomes, selective reporting of data for ICU length of stay was identified. Re-calculation of statistics at higher precision showed that ICU length of stay of patients in the human albumin group was significantly shorter than that of patients in the 6% hydroxyethyl starches 130/0.4 group (standard mean difference − 0.181, 95% confidence interval − 0.361 to − 0.001, P = 0.049), which may offset any proposed economic advantage of using 6% hydroxyethyl starches 130/0.4. At the same time, the renal safety of 6% hydroxyethyl starches 130/0.4 in surgical patients is under regulatory review.

**Conclusions:**

Underpowered trials and selective reporting may impair the validity of the meta-analysis. A more cautious conclusion about the interchangeability between human albumin and 6% hydroxyethyl starches 130/0.4 in cardiac surgery should have been reached.

## Dear editors,

I read with interest the recent publication of a meta-analysis of randomized controlled trials that compared the use of human albumin (HA) solutions with 6% hydroxyethyl starches (HES) 130/0.4 in cardiac surgery (Wei et al. [1]). The meta-analysis included ten studies involving a total of 1567 patients; seven of the studies (579 patients) contained eligible data on total volume of infusion and transfusion frequencies, respectively; six studies (437 patients) exhibited sufficient data on blood loss; length of stay in ICU and hospital were reported in three (478 patients) and four (348 patients) studies, respectively; data on acute kidney injury, the requirement for postoperative renal replacement therapy, and mortality were available from two, two, and three studies, respectively (with less than 345 patients each). Trials were mostly small, single-center studies, and the total number of participants was low, making the meta-analysis underpowered for several outcomes. This was discussed as a limitation of the study because of its potential contribution to trial heterogeneity. Heterogeneity was additionally increased by including both pediatric and adult patients, as well as pump priming and volume resuscitation as colloid indications. Overall, there were no significant differences in effect sizes between HA and HES 130/0.4 reported for any of the endpoints (volume expansion, frequency of transfusions, number of days in ICU and hospital, acute kidney injury, need for renal replacement therapy, and mortality). In the absence of significant differences, the authors concluded that 6% HES 130/0.4 might be a substitute for HA for economic reasons, because HA is more expensive than HES 130/0.4 [1].

Two aspects of this study merit attention. First, a very similar meta-analysis was published in 2014 that compared HES 130/0.4 (tetrastarch, molecular weight 130 kDa, and molar substitution 0.4) with HA and other HES solutions (HES 264/0.45, HES 120/0.5, HES 200/0.5, HES 250/0.5, HES 400/0.7, and HES 450/0.7) [2]. While additional articles on HES 130/0.4 have been published, Wei et al. excluded HES solutions other than 130/0.4, although regulatory authorities recently confirmed that HES safety is independent of molecular weight and substitution [3, 4]. Therefore, it is important to include other HES solutions in the present meta-analytic update.

Second, and more importantly, the reported lack of significant differences in any of the investigated outcome parameters of the meta-analysis may not only be due to low sample sizes, but also be subject to reporting bias. For ICU length of stay after cardiac surgery, Wei et al. [1] reported a standard mean difference of − 0.18 days (95% confidence interval, − 0.36 to 0.00) as not being significantly different (test for overall effect Z = 1.94, P = 0.05) between 235 patients treated with HA and 243 patients treated with HES 130/0.4. However, when recalculating (Comprehensive Meta Analysis Version 2.2.64 software), the overall effect size for ICU length of stay in HA versus 6% HES 130/0.4 group patients using the same fixed-effects model but with higher precision, a different conclusion is reached. More precisely, the standard mean difference of -0.181 (95% confidence interval, -0.361 to -0.001) indicates a significantly reduced ICU length of stay in favor of HA (Z = 1.972, P = 0.049). Comparing HA with HES 130/0.4 in cardiac surgery, the use of HA is associated with significantly reduced ICU length of stay, which may offset the higher acquisition costs of HA. In the present case, the interpretation of statistical results changes, when three instead of two places after the decimal point are shown and used.

Underpowered trials and selective reporting may impair the validity of the meta-analysis’s main conclusion on the interchangeability of HA and HES 130/0.4 solutions in the perioperative fluid management of cardiac surgery patients. To clarify this issue, the results of ongoing surgical trials on 6% HES 130/0.4 [5, 6] will have to be awaited.

## Data Availability

Not applicable.

## Abbreviations

HA: Human albumin
HES: Hydroxyethyl starches
ICU: Intensive care unit
